# Relationship between Viscoelastic Properties of Tissues and Bioimpedance Spectroscopy in Breast-Cancer-Related Lymphedema

**DOI:** 10.3390/jcm11051294

**Published:** 2022-02-26

**Authors:** Alicja Naczk, Janusz Doś, Magdalena Górska-Doś, Robert Sibilski, Piotr Gramza, Ewa Gajewska, Mariusz Naczk

**Affiliations:** 1 Faculty of Physical Culture in Gorzow Wielkopolski, University School of Physical Education in Poznan, Estkowskiego Street 13, 66-400 Gorzow Wielkopolski, Poland; a.naczk@awf-gorzow.edu.pl (A.N.); j.dos@awf-gorzow.edu.pl (J.D.); m.gorska-dos@awf-gorzow.edu.pl (M.G.-D.); 2 Department of Oncological Physiotherapy, Greater Poland Cancer Centre, Garbary Street 15, 61-866 Poznan, Poland; 3 Institute of Medical Sciences, Collegium Medicum, University of Zielona Gora, Licealna Street 9, 65-417 Zielona Gora, Poland; r.sibilski@cm.uz.zgora.pl; 4 Association of Lubusz Innovation Network, Teatralna Street 49, 66-400 Gorzow Wielkopolski, Poland; p.gramza@slsi.pl; 5 Department of Developmental Neurology, Poznan University of Medical Sciences, 49 Przybyszewskiego Street, 60-355 Poznan, Poland; ewagajewska@ump.edu.pl; 6 Institute of Health Sciences, Collegium Medicum, University of Zielona Gora, Licealna Street 9, 65-417 Zielona Gora, Poland

**Keywords:** BCRL, myotonometry, mastectomy, breast cancer, lymphedema

## Abstract

The aim of this study was to assess the relationship between the viscoelastic properties of tissues and breast-cancer-related lymphedema (BCRL). After a mastectomy, 46 females were allocated into a lymphedema group (L; *n* = 15, lymphedema occurred) and a control group (C; *n* = 31, lack of lymphedema). Bioimpedance spectroscopy was used to test BCRL. The mechanical properties of the tissues in both upper limbs were tested using myotonometry. In group L, tone, stiffness, relaxation time, and creep measured on the biceps brachii of the impaired limb significantly differed from the results on the unimpaired limb. In group C, the differences were not significant. Moreover, both tone and stiffness were inversely correlated with the level of lymphedema (r = −0.72 and r = −0.88, respectively), and both relaxation and creep were significantly related to the level of lymphedema (r = 0.71 and r = 0.59, respectively), when myotonometry was completed on the biceps brachii of the impaired limb in group L. The relationships were not significant in group C. Measurements of viscoelastic properties can provide useful information concerning lymphedema. Our findings suggest that significant correlations between selected mechanical properties of the tissues and BCRL can be used in BCRL detection and treatment.

## 1. Introduction

Breast cancer accounts for over 24% of all cancers among females [1]. One of the many complications of breast cancer treatment is lymphedema of the upper extremity, a chronic condition, which can be a challenge to both patients and clinicians. Various studies demonstrate a wide variety of incidence rates in which approximately 16.6% of breast cancer survivors develop lymphedema [2]. However, Fu et al. [3] stated that up to 40% of females treated for breast cancer had lymphedema. Breast cancer survivors with lymphedema in the upper extremity report symptoms, including pain, heaviness, stiffness, tightness, and a decreased range of motion. Moreover, quality of life and daily function significantly deteriorate [4,5,6]. A quick and accurate diagnosis of breast-cancer-related lymphedema (BCRL) is needed for breast cancer survivors after mastectomy. Volumetric measures, including those derived from girth measurements, perometry, or water displacement, are used to screen for arm volume changes indicative of lymphedema. However, these methods have some limitations: they do not provide information about tissue properties, and changes in muscle mass and fat mass can lead to wrong conclusions. Moreover, bioelectrical impedance analysis (BIA) is used to detect lymphedema [2]. Cornish et al. [7] stated that multiple-frequency BIA is an extremely sensitive and reliable technique for the early detection of lymphedema. However, it has limitations when assessing longstanding lymphedema and changes in tissues, as the tissue properties may be more fatty and fibrotic. Therefore, in our opinion, new methods of BCRL detection and treatment should be developed.

One of the methods that could potentially be used in the diagnosis of BCRL is the assessment of the viscoelastic properties of tissues (myotonometry). Myotonometry is becoming increasingly popular in assessing the viscoelastic properties of muscles and skin in many diseases. MyotonPRO (Myoton AS, Estonia) has been used in medicine to evaluate disease detection, progression, and treatment efficacy. Research shows that MyotonPRO can be used to evaluate sclerosis in chronic graft-versus-host disease [8], cerebral palsy [9,10], spinal cord injury [11], and stroke [12]. A myotonometer is a portable, handheld device that non-invasively and objectively measures the mechanical properties of muscles. It percutaneously applies a short, constant force impulse to the muscle by a probe, producing oscillations of the muscle. A built-in accelerometer measures tissue oscillations and tissue properties; tone, stiffness, elasticity, relaxation, and creep are simultaneously calculated. The accumulation of excess protein-rich lymph fluid causes abnormal swelling in the upper extremity and can alter the resistance to pressure and the elasticity of skin and subcutaneous tissues, which is related to a patient’s perception of hardness in the upper extremity afflicted with lymphedema [13]. Therefore, it can be hypothesized that changes in the elasticity of skin and subcutaneous tissues can be detected by myotonometry, which allows the evaluation of the viscoelastic properties of tissues.

To date, there are no data on the use of myotonometry in BCRL diagnostics and treatment, and this is an exploratory study. Taking into consideration current knowledge about BCRL and myotonometry, it was hypothesized that interstitial accumulation of protein-rich lymphatic fluid under the skin will lead to changes in skin stiffness and elasticity. Thus, lymphedema can result in different stiffness and elasticity of tissues in the impaired limb compared to the healthy limb. The present study aimed to assess the relationship between the viscoelastic properties of tissues and bioimpedance spectroscopy (BIS) in breast-cancer-related lymphedema. In our opinion, it can be a first step to assess the usefulness of myotonometry in the diagnosis of BCRL.

## 2. Materials and Methods

### 2.1. Participants

The study included 46 female breast cancer survivors who had experienced a mastectomy (mean ± standard deviation: age, 67.2 ± 11.3 years, range 43–82 years; body mass, 71.9 ± 11.1 kg; height, 159 ± 5.6 cm). The participants were members of the Amazons Association, an association for individuals who have experienced a mastectomy (average time from surgery: 12.1 years). All participants met the inclusion criteria: a mastectomy was performed at least six months before the study and received permission from their physician to participate in the experiment. The exclusion criteria included serious diseases of the heart, kidneys, or liver, cerebral palsy, Parkinson’s disease, and limb amputations. Among the participants, 30 had undergone a simple mastectomy, and 16 had undergone breast-conserving surgery (partial mastectomy); more details are shown in Table 1.

The participants were allocated into two groups, a lymphedema group (L; *n* = 15, occurred) and a control group (C; *n* = 31, lack of lymphedema), based on an evaluation of edema using BIA and an L-Dex device. According to Fu et al. [3], the recommendations suggested the cut-off point for an L-Dex ratio > +7.1. Therefore, all patients with an L-Dex index > +7.1 were placed in group L, and the other patients were placed in group C. Moreover, using the guidelines of the International Society of Lymphology [14], it was estimated that within group L, four women were in early (stage I) lymphedema, seven in stage II, and four in stage III. All participants provided written informed consent to take part in the study. All procedures were approved by the Ethics Committee of Poznan University of Medical Science in Poland, with approval based on the Declaration of Helsinki.

### 2.2. Body Composition Measurements

We performed BIAs to evaluate the body composition of participants (Tanita 980 MC, Japan). The participants were asked to maintain a normal state of hydration, and they were not allowed to exercise, eat, or drink alcohol or caffeine for 12 h preceding the measurements. Measurements were conducted in the morning, according to the manufacturer’s guidelines. Fat mass, muscle mass, and water content measurements were collected for analyses.

#### BCRL Evaluation

Bioimpedance spectroscopy was used to evaluate BCRL. This technique allows the detection of small changes in extracellular fluid and subclinical BCRL. Thus, it provides subclinical detection of BCRL when swelling is not apparent. Measurements were performed using an L-Dex U400 unit (ImpediMed Limited, Pinkenba, Australia). The feasibility and clinical utility of implementing L-Dex measurements in routine breast cancer care have been confirmed in previous studies [15,16]. The patients were measured using a standardized technique that required them to lay supine on a non-metallic surface [15].

### 2.3. Measurement of Mechanical Parameters

A handheld MyotonPRO device (Myoton AS, Estonia) was applied to measure the viscoelastic properties of tissues. The probe of MyotonPRO, placed on the skin’s surface, produced a short mechanical impulse. Oscillations due to the response of soft tissues were then processed by the device. It calculated five parameters: tone, stiffness, elasticity, relaxation, and creep. Detailed specifications of the device and a description of calculated parameters were presented by Schneider et al. [17]. MyotonPRO reproducibility was tested in many studies, and authors concluded that this device is a reliable tool for quantifying tissue properties both in healthy young people and in subjects suffering from various diseases [18,19,20].

**Figure 1:**
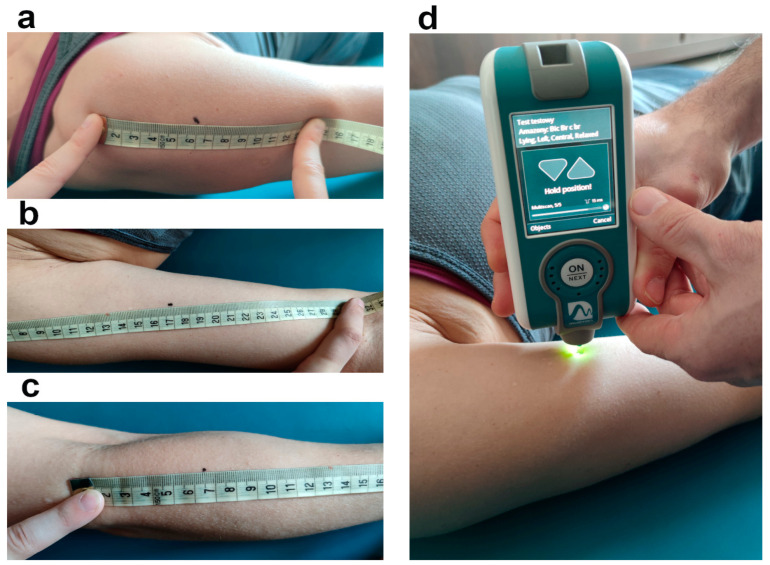
The location of testing points for tested muscles: (**a**) deltoid; (**b**) biceps brachii; (**c**) brachioradialis; (**d**) measurement of the viscoelastic properties of tissues using Myoton.

All outcomes were obtained with the participant in a supine position, the upper extremities placed along the trunk, and the forearms placed in a position midway between supination and pronation. The participants were asked to fully relax for two minutes before the measurement was taken. Measurement points were marked on the skin with a non-toxic washable ink pen. The location of testing points for tested muscles are presented on Figure 1a–c and included:

- Deltoid: the central part of the deltoid muscle in the middle part of the muscle belly;
- Biceps brachii: the equidistant point between the anterior aspect of the lateral tip of the acromion and the medial border of the cubital fossa;
- Brachioradialis: the central part of the brachioradialis muscle in the middle part of the muscle belly.

When the probe of MyotonPRO was correctly positioned (perpendicular to the skin’s surface), a green light appeared, and the device generated five mechanical impulses through the end of the small lever. For each measurement point, three measurements were made (fifteen mechanical impulses for each measurement point); Figure 1d.

### 2.4. Statistical Analysis

Data normality was tested using the Shapiro–Wilk test. Descriptive statistics were expressed as the mean ± standard deviation. Lower and upper limits of the 95% confidence intervals (95% CI) for selected variables were calculated. For evaluating the significance of changes, an analysis of variance (ANOVA) was performed. When differences were detected, the Scheffé post hoc procedure was applied to determine where the differences occurred. Pearson correlation coefficients were determined for selected variables. The level of significance was set at *p* ≤ 0.05.

## 3. Results

BCRL assessed by the L-Dex index was significantly higher in group L (22.4 ± 18.0; 95% CI [13.3 to 31.5]) than in group C (−0.53 ± 3.91; 95% CI [−1.91 to 0.85]). Moreover, in group L, tone, stiffness, relaxation time, and creep measured on the biceps brachii of the impaired limb significantly differed from those obtained on the unimpaired limb. A comparison of the mechanical properties of the tissues in the impaired and unimpaired limbs measured on the deltoid and brachioradialis in group L showed that they did not significantly differ (Table 2). However, differences in stiffness measured on the deltoid and brachioradialis in the impaired and unimpaired limbs were close to significance (*p* = 0.0503 and 0.06, respectively). In group C, the mechanical properties of the tissues did not significantly differ, regardless of the place of measurement (Table 2).

The percentage differences in tone, stiffness, relaxation, and creep between limbs within the groups significantly differed between the groups (Table 3).

**Figure 2:**
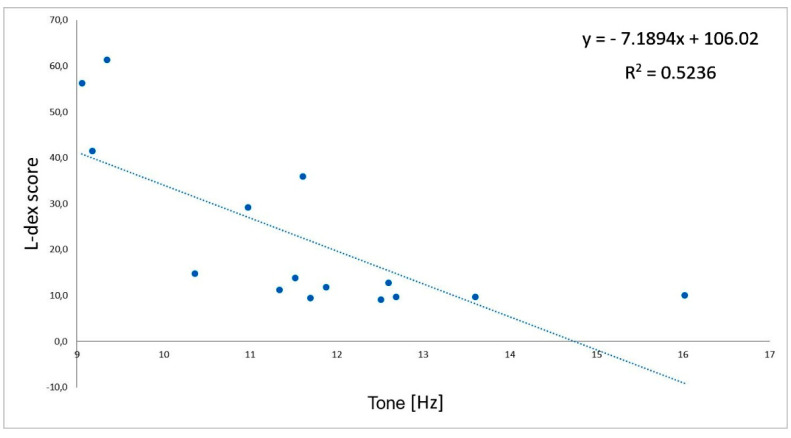
Correlations between the L-Dex index and tone (oscillation frequency) measured using MyotonPRO.

**Figure 3:**
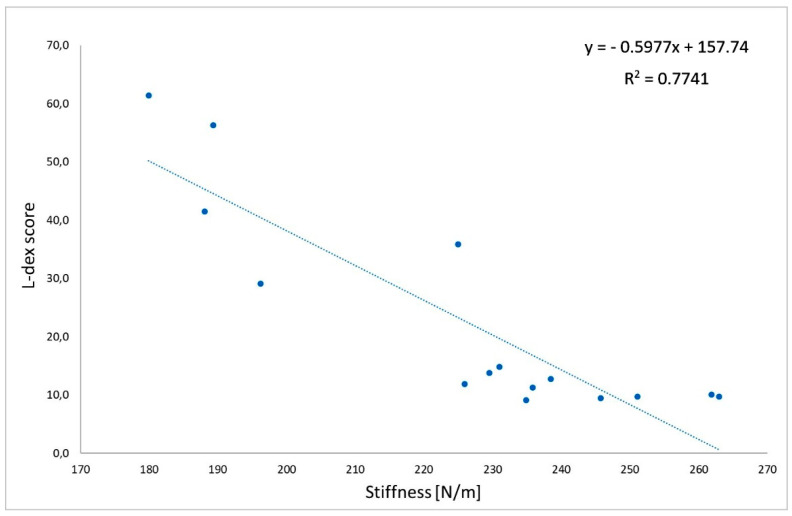
Correlations between the L-Dex index and stiffness measured using MyotonPRO.

**Figure 4:**
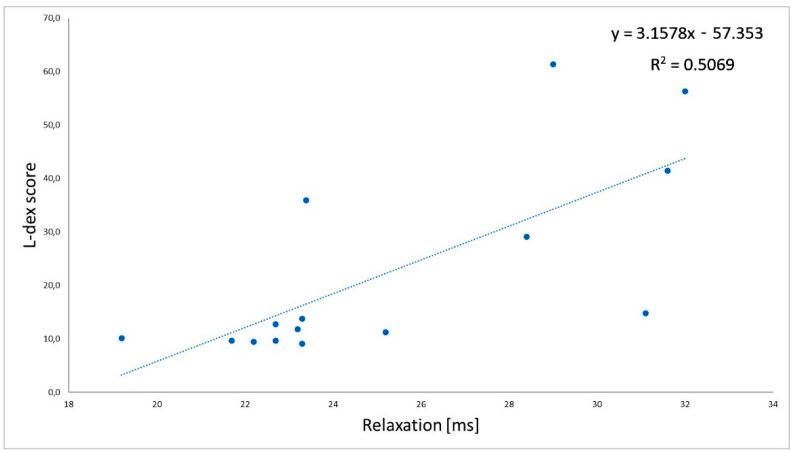
Correlations between the L-Dex index and relaxation measured using MyotonPRO.

**Figure 5:**
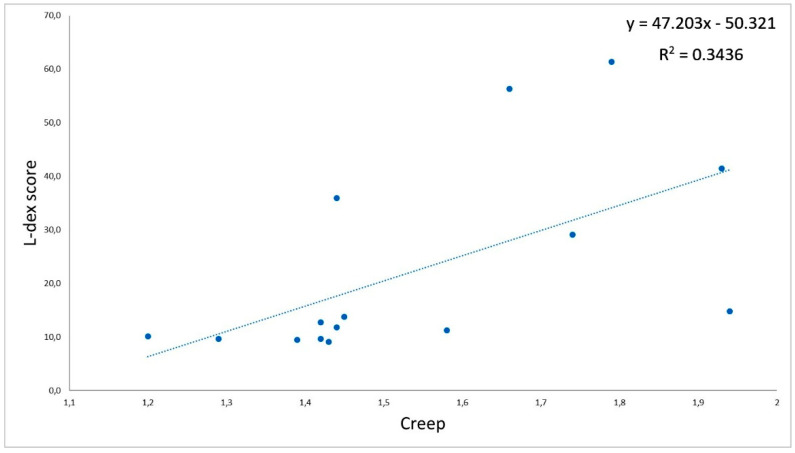
Correlations between the L-Dex index and creep measured using MyotonPRO.

There were negative, significant correlations between the L-Dex index and tone (r = −0.72; Figure 2) and between the L-Dex index and stiffness (r = −0.88; Figure 3) measured on the biceps brachii of the impaired limb in group L. Moreover, there were positive, significant correlations between the L-Dex index and relaxation (r = 0.71; Figure 4) and between the L-Dex index and creep (r = 0.59; Figure 5) measured on the biceps brachii of the impaired limb in group L.

The correlations calculated for group L between the L-Dex index and the mechanical properties of the tissues measured on the deltoid and brachioradialis of the impaired limb were not significant. Moreover, the correlations for group L between the L-Dex index and the mechanical properties of the tissues measured on the unimpaired limb were not significant, regardless of the place of measurement. The correlations calculated for group C between the L-Dex index and the mechanical properties were not significant, regardless of the place of measurement.

There were no significant differences in body composition between the groups (Table 4). However, there were significant correlations between the L-Dex index and body mass index—BMI (r = 0.57), fat (%) (0.57), and fat (kg) (0.54) in group L, whereas there were no significant correlations between these parameters in group C.

## 4. Discussion

Significant differences in the mechanical properties between impaired and unimpaired limbs in group L were noted when the measurements were taken from the biceps brachii. The different results were probably a result of the accumulation of lymph under the skin of the impaired limb. It is very likely that the lymph accumulation led to decreased tissue tension and stiffness and an increase in time for the tissue to restore its shape from deformation.

It is possible that a significant correlation between values of the L-Dex index and the mechanical properties of the biceps brachii noted in group L indicates that myotonometry can be used in BCRL detection. Moreover, it seems that myotonometry can be used to test the treatment efficacy of BCRL. It is consistent with the conclusion in Schneider et al. [17] that changes in muscle tone and properties could be used to assess the effects of a therapeutic intervention. The authors added that such assessments could be performed at regular intervals to monitor the stage of the pathological processes of muscles and for assessing the efficacy of a therapeutic intervention. Based on the results, it is recommended to measure the mechanical properties of the biceps brachii of both impaired and unimpaired limbs. If differences appear, it can suggest that BCRL is developing. The differences in tone, stiffness, relaxation, and creep between limbs noted in group L significantly differed (the differences were always higher than 20% in group L), while in group C, the differences between limbs were not significant (always lower than 3%). The percentage differences in tone, stiffness, relaxation, and creep between limbs within the groups significantly differed. Therefore, if after a mastectomy procedure, the biceps’ stiffness, tone (frequency), relaxation, and creep noted in the impaired limb differs more than 20% from the properties observed in the unimpaired limb, BCRL may be indicated. Moreover, if the difference is greater than 5%, there is a high risk of developing BCRL. However, further research is needed to define these criteria precisely.

We believe that myotonometry can be used in the detection of lymphedema; in our opinion, the most valuable measurement index is the dynamic stiffness of tissues. Dynamic stiffness is the biomechanical property of tissue that characterizes the resistance to an external force that deforms its initial shape—the higher the value, the higher the stiffness. When the impulse force is applied to the tissue (Myoton probe generated force 0.4 N in time 15 ms under constant pre-compression force 0.18 N), the tissue deforms. If the tissue is hard (small amount of extracellular fluid), its resistance to an external force is greater, and it quickly returns to its initial shape after force application—the tissue has a high dynamic stiffness. If more lymph accumulates under the skin, the resistance of the tissue to an external force is smaller—the deformation of the tissue is greater, and its stiffness is lower. Moreover, the tissue needs more time to return to its initial shape, so the relaxation time is longer.

It is worth noting that the dominance of the limb may affect the results obtained during myotonometric measurements. Studies by Alvarez-Diaz et al. [21] have shown that some muscles of the lower limbs (rectus femoris, vastus medialis, and vastus lateralis) differ in mechanical parameters between the dominant and non-dominant limbs. Moreover, Aird et al. [22] reported that stiffness tested in the elderly was not statistically different between the dominant and non-dominant limbs, but the differences were close to significant (rectus femoris *p* = 0.071). On the other hand, Bailey et al.’s studies [23] showed no significant differences in the biceps brachii stiffness between the dominant and non-dominant limbs (235.0 N/m vs. 238.6 N/m, respectively) in the elderly. Similar conclusions were presented by Pappas et al. [24] (young men were tested); lower-limb muscle stiffness did not significantly differ between dominant and non-dominant limbs.

We did not note a significant difference in body composition between both groups. However, a significant correlation between the L-Dex index and BMI and fat in group L was found, whereas there were no significant correlations between these parameters in group C. This suggests that a high BMI and high content of body fat promote the development of BCRL. Similar conclusions were presented by Johansson et al. [25] and Ayre and Parker [26], who stated that a higher BMI before and after operation increases the lymphedema risk. However, it should be noted that the clinical manifestations of lymphedema are secondary to an inflammatory response to the chronic accumulation of protein-containing interstitial fluid and adipose tissue. Lymph stasis, or decreased flow, has been shown to contribute to lipogenesis and fat deposition, which later leads to increased fibrocyte activation and connective tissue growth [25]. Therefore, the appearance of lymphedema and the amount of adipose tissue are strongly correlated. It was interesting for us to check correlations between the BIA and BIS results. Our results indicated that there were no significant correlations between extracellular water volume (ECW; noted during BIA) and the L-Dex index (BIS). Other conclusions were found by Yasunaga et al. [27], who noted that the %ECW value tested by BIA might be a simple and useful indicator of the development and severity of leg lymphedema. In our study, we used the Tanita 980 MC BIA analyzer, which assesses the global ECW, while the Yasunaga et al. studies [27] used the InBody S10 analyzer, which allows the user to determine the ECW in different body segments, including each limb separately. BIA may also be useful for assessing the reductive effect of lymphaticovenular anastomosis on upper-limb lymphedema [28]. Thus, it is possible that BIA can provide useful information on lymphoedema if the ECW is evaluated using an appropriate analyzer.

### Limitations of the Study

The main study limitation was the small group of participants, which restricted our ability to draw strong conclusions. A larger group of participants would allow for a more precise determination of the borderline values of the myotonometry parameters indicating lymphedema. Moreover, we did not evaluate lymphedema using other methods than bioimpedance spectroscopy. Future research should also determine the usefulness of myotonometry for the detection of lymphoedema, depending on the phase of its development. Finally, preoperative measurements were not performed. In our opinion, this study is a first step to assess the usefulness of myotonometry in the diagnosis of BCRL; however, no strong conclusions can be made about the usefulness of myotonometry in the diagnosis of BCRL.

## 5. Conclusions

The results of our study showed significant correlations between the mechanical properties of biceps brachii tissues measured in the impaired limb and the L-Dex in women with BCRL. These correlations may suggest that myotonometry can be used in BCRL detection. Measurements of the viscoelastic properties of the central part of the biceps brachii of impaired and unimpaired limbs can provide useful information concerning lymphedema. Moreover, it is possible that myotonometry can be useful for the evaluation of lymphedema development and treatment efficacy of BCRL; however, future studies are needed.

## Figures and Tables

**Table 1:** Treatment applied in lymphedema group (L) and control group (C).

Group	Simple Mastectomy	Breast-Conserving Surgery	Radiotherapy	Chemotherapy	Hormone Therapy	Lymphadenectomy
L	11	4	10	12	11	14
C	19	12	15	15	21	21

**Table 2:** Mechanical properties of tissues measured using MyotonPRO.

		L Group		Control Group	
		Impaired Limb	Unimpaired Limb	Impaired Limb	Unimpaired Limb
Muscle	Parameter	Mean SD		Mean SD	
DL	tone	24.4 ± 4.49	26.0 ± 3.15	23.6 ± 3.82	23.5 ± 4.01
	stiffness	529 ± 131	611 ± 83.7	524 ± 122	523 ± 126
	elasticity	1.37 ± 0.21	1.35 ± 0.29	1.37 ± 0.22	1.39 ± 0.27
	relaxation time	10.2 ± 4.30	8.63 ± 1.65	10.6 ± 3.06	10.5 ± 3.15
	creep	0.66 ± 0.26	0.57 ± 0.10	0.66 ± 0.17	0.67 ± 0.19
BB	tone	11.6 ± 1.81 *	14.8 ± 2.54	13.5 ± 1.95	13.6 ± 1.83
	stiffness	226 ± 26.5 *	282 ± 44.2	256 ± 40.5	249 ± 38.8
	elasticity	2.07 ± 0.31	2.02 ± 0.34	1.79 ± 0.25	1.80 ± 0.28
	relaxation time	25.3 ± 4.06 *	19.9 ± 4.06	22.7 ± 4.47	23.0 ± 4.11
	creep	1.54 ± 0.22 *	1.23 ± 0.24	1.40 ± 0.26	1.41 ± 0.23
BR	tone	15.6 ± 1.57	16.6 ± 1.78	17.0 ± 1.60	16.9 ± 1.37
	stiffness	278 ± 33.7	304 ± 41.0	308 ± 41.3	302 ± 33.9
	elasticity	1.46 ± 0.23	1.38 ± 0.19	1.35 ± 0.25	1.36 ± 0.28
	relaxation time	19.7 ± 3.15	17.9 ± 2.39	17.6 ± 2.37	17.7 ± 2.27
	creep	1.22 ± 0.19	1.11 ± 0.15	1.09 ± 0.14	1.09 ± 0.14

DL—deltoid, BB—biceps brachii. BR—brachioradialis; *—significant difference between impaired and unimpaired limb within group.

**Table 3:** Percentage differences in mechanical properties measured on the biceps brachii of impaired and unimpaired limbs within groups.

Group/Parameter	Tone	Stiffness	Elasticity	Relaxation Time	Creep
L	27.6 ± 15.3 *	26.1 ± 24.3 *	−1.07 ± 17.4	−21.2 ± 13.5 *	−20.1 ± 12.4 *
C	1.45 ± 13.4	−1.73 ± 13.8	1.86 ± 17.5	2.83 ± 16.1	2.75 ± 15.6

*—significant difference between groups.

**Table 4:** Body composition of participants.

Group	BMI	Fat Mass (%)	Fat Mass (kg)	Total Body Water (kg)
L	29.5 ± 3.10	36.3 ± 3.68	27.2 ± 4.01	33.5 ± 2.75
C	28.4 ± 4.04	37.6 ± 3.99	27.3 ± 7.35	31.3 ± 3.98

## Data Availability

The data presented in this study are available on request from the corresponding author.
